# Short Chain (≤C4) Esterification Increases Bioavailability of Rosmarinic Acid and Its Potency to Inhibit Vascular Smooth Muscle Cell Proliferation

**DOI:** 10.3389/fphar.2020.609756

**Published:** 2021-01-21

**Authors:** Tina Blažević, Gottfried Reznicek, Limin Ding, Gangqiang Yang, Patricia Haiss, Elke H. Heiss, Verena M. Dirsch, Rongxia Liu

**Affiliations:** ^1^ Department of Pharmacognosy, Faculty of Life Sciences, University of Vienna, Vienna, Austria; ^2^ School of Pharmacy, Key Laboratory of Molecular Pharmacology and Drug Evaluation, Ministry of Education, Collaborative Innovation Center of Advanced Drug Delivery System and Biotech Drugs in Universities of Shandong, University of Yantai, Yantai, China

**Keywords:** vascular smooth muscle cells, rosmarinic acid, proliferation, pharmacokinetics, rosmarinic acid esters

## Abstract

Rosmarinic acid is a natural phenolic acid and active compound found in many culinary plants, such as rosemary, mint, basil and perilla. Aiming to improve the pharmacokinetic profile of rosmarinic acid and its activity on vascular smooth muscle cell proliferation, we generated a series of rosmarinic acid esters with increasing alkyl chain length ranging from C1 to C12. UHPLC-MS/MS analysis of rat blood samples revealed the highest increase in bioavailability of rosmarinic acid, up to 10.52%, after oral administration of its butyl ester, compared to only 1.57% after rosmarinic acid had been administered in its original form. When added to vascular smooth muscle cells *in vitro*, all rosmarinic acid esters were taken up, remained esterified and inhibited vascular smooth muscle cell proliferation with IC_50_ values declining as the length of alkyl chains increased up to C4, with an IC_50_ of 2.84 µM for rosmarinic acid butyl ester, as evident in a resazurin assay. Vascular smooth muscle cells were arrested in the G_0_/G_1_ phase of the cell cycle and the retinoblastoma protein phosphorylation was blocked. Esterification with longer alkyl chains did not improve absorption and resulted in cytotoxicity in *in vitro* settings. In this study, we proved that esterification with proper length of alkyl chains (C1–C4) is a promising way to improve *in vivo* bioavailability of rosmarinic acid in rats and *in vitro* biological activity in rat vascular smooth muscle cells.

## Introduction

Blood vessel wall disorders like atherosclerosis and restenosis are characterized by a switch of quiescent vascular smooth muscle cells (VSMC) into a proliferative and synthetic phenotype. Platelet-derived growth factor (PDGF) is a powerful stimulator of VSMC migration and proliferation. Identification of compounds counteracting the PDGF-induced VSMC proliferation would be an effective approach for ameliorating these vessel wall disorders (Levitzki, 2004).

Aside from having nutritional value, food and spices are excellent sources for lead structure identification. We previously found that polyphenols from Mediterranean spices could inhibit VSMC proliferation, especially rosmarinic acid (RA) and its congeners. Among the 12 tested constituents, rosmarinic acid methyl ester (RAME) showed the best anti-proliferative activity in VSMC, even more potent than RA, and it inhibited neointima formation *in vivo* (Liu et al., 2018).

**FIGURE 1:**
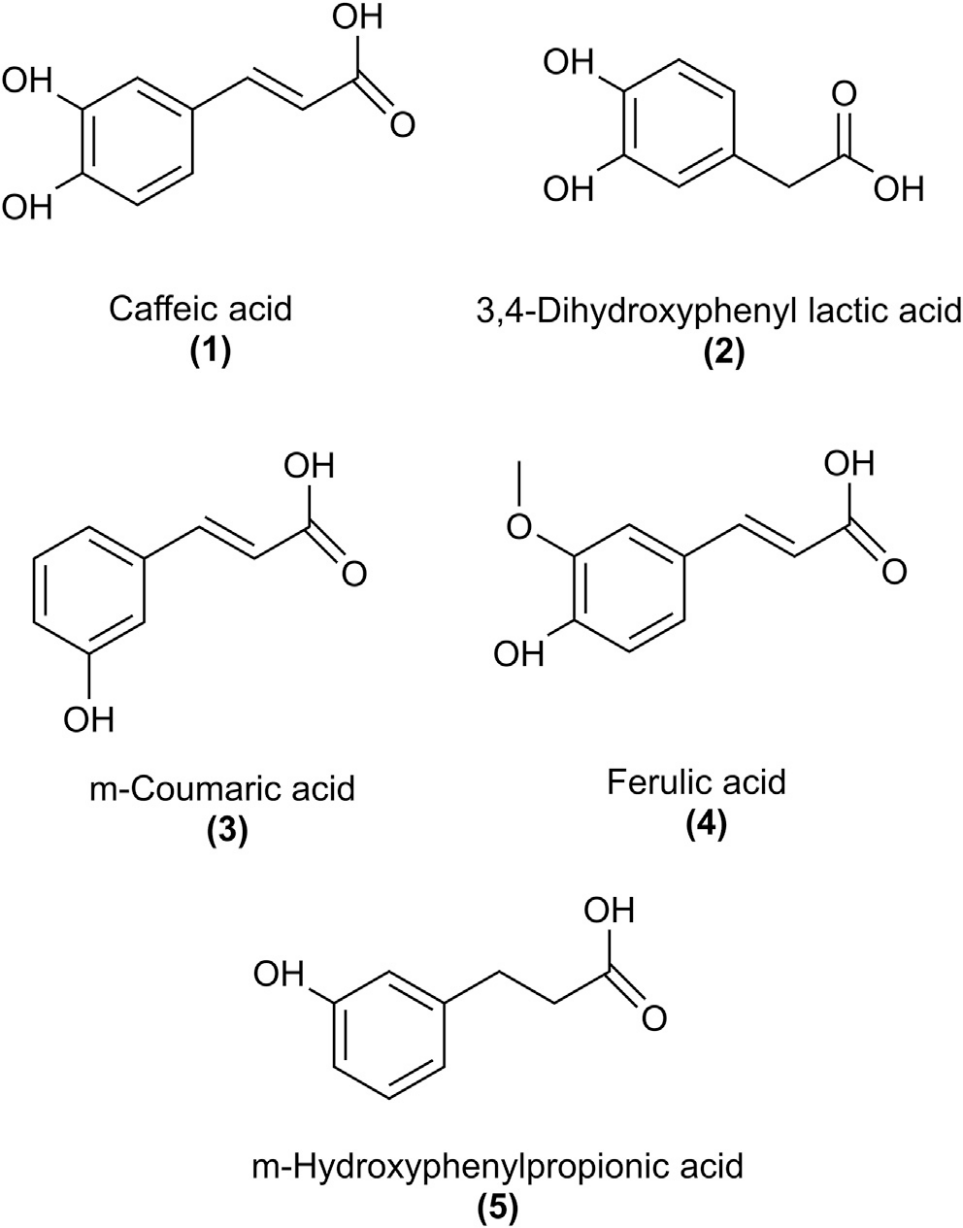
Structures of rosmarinic acid metabolites that were examined for the inhibition of PDGF-BB-induced VSMC proliferation.

RA is an ester of caffeic (1) and 3,4-dihydroxyphenyl lactic acid (5). Besides chlorogenic acid, it represents one of the most frequently occurring caffeic acid esters in the whole plant kingdom (Petersen, 2013). The most prominent plant families containing RA are *Boraginaceae* and *Lamiaceae* (sub-family *Nepetoideae*). RA is an integral part of the daily human diet, found as well in food supplements to act preventive or therapeutic against various diseases. It shows extensive pharmacological activities, e.g. antioxidant, anti-inflammatory, antiviral and cardioprotective (Nunes et al., 2017). However, poor oral bioavailability and marked metabolism impeded exploitation of RA as a therapeutic agent. Different pharmacokinetic studies in rats and humans showed that only a small amount of orally ingested RA, up to 1.69%, is absorbed, presumably in the upper intestine (Nakazawa and Ohsawa, 1998; Baba et al., 2005; Wang et al., 2017), and in part in its conjugated form (Baba et al., 2005; Noguchi-Shinohara et al., 2015). Similar reports on low bioavailability of RA were obtained from absorption studies using the Caco-2 cell model (Qiang et al., 2011; Villalva et al., 2018). Unabsorbed RA seems to reach the colon, where it is hydrolyzed by gut bacteria into metabolites, like caffeic acid (1) and 3,4-dihydroxyphenyl lactic acid (2) (Nakamura et al., 1998; Bel-Rhlid et al., 2009; Zoric et al., 2016) as depicted in Figure 1. Other metabolites were also reported after ingestion of RA in rat and human studies, like intact and conjugated forms of methylated RA (Baba et al., 2005), *m*-coumaric acid (3), ferulic acid (4) (Nakazawa and Ohsawa, 1998; Baba et al., 2005) and *m*-hydroxyphenylpropionic acid (5) (Nakazawa and Ohsawa, 1998; Mosele et al., 2014). Cumulative proportions of all RA metabolites detected in urine corresponded to about 32% and 6% of the administered dose in rat (Nakazawa and Ohsawa, 1998) and human (Baba et al., 2005) studies, respectively. However, to what extent so far identified metabolites account for the biological effects of RA has been largely underexplored.

The high hydrophilicity is one of the main reasons for RA's poor bioavailability. Ester-containing small molecules are common prodrugs, as exemplified by acetylsalicylic acid (aspirin). Esterification of plant polyphenols can ameliorate their strong resistance to 1st-pass effects during absorption, as shown in the case of quercetin (Biasutto et al., 2007; Hu et al., 2016).

**FIGURE 2:**
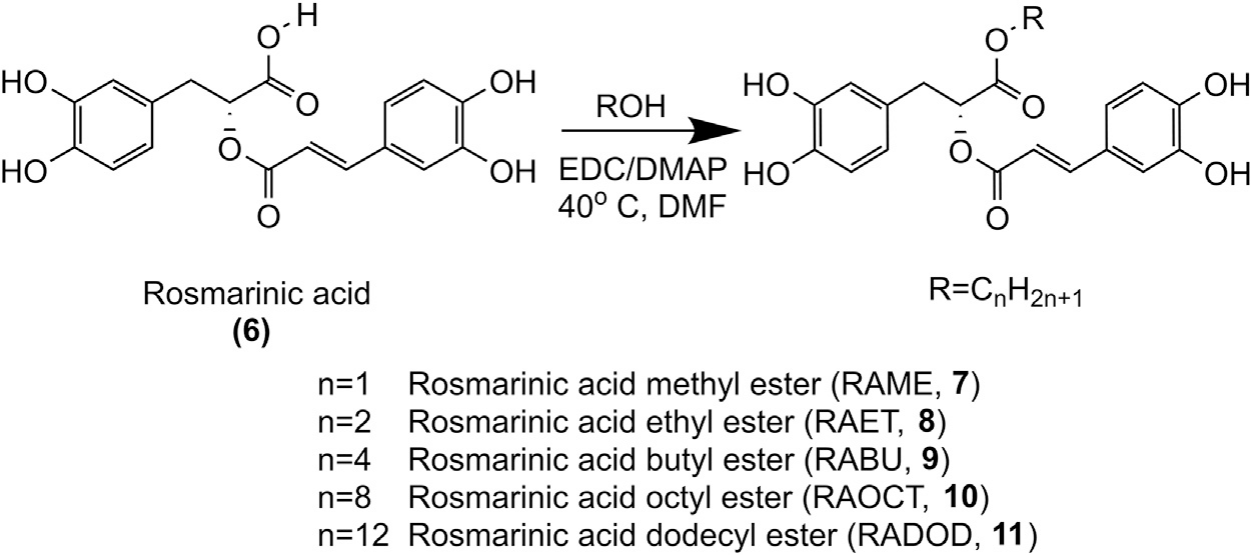
Synthesis of methyl, ethyl, butyl, octyl and dodecyl RA esters.

Prompted by our previous findings that the methyl-ester of RA was more potent against PDGF-induced VSMC proliferation than RA (Liu et al., 2018), we hypothesized that esters of RA may serve as prodrugs with increased cell permeability *in vitro*, higher bioavailability *in vivo* and subsequent higher intracellular levels of bioactive RA. A series of RA (6) alkyl esters was synthesized: methyl (7), ethyl (8), butyl (9), octyl (10) and dodecyl (11), as shown in Figure 2, and the effect of the alkyl chain length on bioavailability of RA *in vivo* as well as cell permeability and antiproliferative activity in VSMC *in vitro* were investigated. Moreover, known RA metabolites were tested for inhibition of VSMC growth *in vitro*.

## Materials and Methods

### Materials

Rosmarinic acid, caffeic acid, ferulic acid, *m*-coumaric acid and 3,4-dihydroxyphenyl lactic acid were purchased from Victory Biological Technologies (Sichuan, China, purity of compounds as stated by the company was >95%), whereas *m*-hydroxyphenylpropionic acid (purity obtained by HPLC was 99.8%, as stated by the company’s COA) was from Sigma-Aldrich (MO, United States). Methyl, ethyl, butyl, octyl and dodecyl RA esters were synthesized in our laboratory (Key Laboratory of Molecular Pharmacology and Drug Evaluation, University of Yantai, China), and their structures were confirmed by high-resolution MS data and NMR spectroscopic data (see Supplementary Material). The purity of synthesized compounds was analyzed on Waters Acquity I-Class UHPLC system, using chromatographic column Waters BEH C18 (2.1 × 50 mm, 1.7 µm) and water (V:V; 0.1% formic acid) as mobile phase A and acetonitrile (V:V; 0.1% formic acid) as mobile phase B. Elution was conducted under a flow rate of 0.3 ml/min and conditions as follows: 0–1 min, 5%–15% B; 1–3 min, 15% B; 3–3.5 min, 15%–25% B; 3.5–4 min, 25% B; 4–4.5 min, 25%–40% B; 4.5–6 min, 40%–70% B; 6–7 min, 70% B; 7–7.5 min, 70%–95% B; 7.5–8 min, 95% B; 8–8.1 min, 95%–5% B; 8.1–13 min, 5% B. The absorption was detected at 330 nm. The determined purities of RA esters were: 98.68% for methyl-, 96.82% for ethyl-, 95.46% for butyl-, 96.46% for octyl- and 97.86% for dodecyl-ester, and the respective chromatograms are depicted in Supplementary Figure S1.

Silibinin, used as an internal standard (IS), was obtained from the National Institute for the Control of Pharmaceutical and Biological Products (Beijing, China). The purities of all reference standards were determined to be over 95% by HPLC-UV. HPLC-grade reagents were obtained from Fisher Scientific (Fairlawn, NJ).

Primary rat aortic VSMC, growth media, and cell culture supplements were purchased from Lonza (Basel, Switzerland). Serum for cell culture was supplied from Gibco Life Technologies (Darmstadt, Germany), and PDGF-BB was obtained from Bachem (Weilheim, Germany).

All other used reagents and chemicals were of analytical grade and obtained from Sigma–Aldrich (MO, United States). The monoclonal anti-phospho-Rb (Ser807/811), the anti-α/β-tubulin and the secondary horseradish-peroxidase-coupled antibody were all from Cell Signaling (Leiden, Netherlands).

### Synthesis of Rosmarinic Acid Alkyl Esters

The synthesis of RAME (7), RA ethyl ester (RAET, 8), RA butyl ester (RABU, 9), RA octyl ester (RAOCT, 10) and RA dodecyl ester (RADOD, 11) were carried out by esterification of RA (6) with corresponding alcohols (methanol, ethanol, n-butanol, n-octanol, n-dodecanol) respectively, as shown in Figure 2. 1-(3-dimethylaminopropyl)-3-ethylcarbodiimide (EDC) and 4-dimethylamino-pyridine (DMAP) were used as catalysts, DMF was the solvent. The products were purified by liquid-liquid extraction and column chromatography. The structures of RA alkyl esters were confirmed by high-resolution MS data and NMR spectroscopic data. High-resolution MS data was recorded on a Q Exactive Orbitrap MS system at a resolution of 70,000 FWHM (Thermo scientific, Waltham, MA, United States). 1H NMR data was measured with a Bruker AVANCE 400 NMR spectrometer at 400 MHz (Bruker, Fallanden, Switzerland). Mass errors obtained from high-resolution MS data of these RA esters were all within 5 ppm (Supplementary Figure S2). 1H NMR data (Supplementary Figures S3–S7) obtained from methyl, ethyl, butyl, octyl and dodecyl RA esters was consistent with those previously reported (Hamada and Abdo, 2015; Wicha et al., 2015; Thammason et al., 2018).

### Bioavailability and Pharmacokinetics of Rosmarinic Acid and Its Esters in Rats

#### Animal Experiment

Male Sprague–Dawley rats (220–250 g) were purchased from Jinan Peng Yue Experimental Animal Breeding Co., Ltd (Shandong, China). Rats were housed under standard conditions with free access to food and deionized water. All animal experimental protocols were approved by the Ethics Committee of Yantai University (IACUC No. 2018-DA-12) and conducted according to the Care and Use of Laboratory Animals of the National Institutes of Health (NIH). Rats were fasted overnight with free access to water before animal experiments. Animals were randomly divided into twelve groups (three rats per group). All compounds were administered as a single dose orally (80 μmol/kg) and as an intravenous injection via tail vein (1 μmol/kg). Compounds were suspended in DMSO: 1% CMC-Na (5:95, v/v) for oral administration and dissolved in DMSO: 0.5% tween 80 (5:95, v/v) as a clear solution for intravenous administration, respectively. Approximately 250 µL blood samples were collected from the orbital veins in heparinized tubes before and 0.033, 0.083, 0.167, 0.25, 0.5, 1, 2, 4, 8 h after drug administration. Blood samples were centrifuged at 8,000 rpm, 4°C for 10 min, and plasma was collected and stored at −20°C until analysis.

#### Sample Preparation

Plasma sample (50 µl) was mixed with 5 µl internal standard solution (1 μg/ml silibinin in methanol) and 145 µl 0.05% formic acid-methanol for protein precipitation. After vortexing for 30 s, the mixture was centrifuged at 13,000 rpm, 4°C for 10 min, and 5 μl of supernatant were injected into UHPLC-MS/MS for analysis.

#### Preparation of Calibration Standards, and Quality Control (QC) Solutions

The stock solution of RA (5 mg/mL) was prepared in methanol and further diluted to 20 μg/ml with water: methanol (1:1, v/v, 0.1% formic acid). The stock solution of IS (silibinin, 2 mg/mL) was prepared in DMSO and further diluted to 1 μg/mL with methanol. Both stock solutions were stored at −20°C before analysis. The working solution of RA was obtained by serial dilutions of stock solution with water: methanol (1:1, v/v, 0.1% formic acid). Then, 5 µl working solutions were added to 45 µl blank plasma and acquired final plasma concentrations at 1, 5, 10, 50, 100, 500, 1,000, 2000 ng/mL for the calibration standards and 2, 40, 800 ng/mL for QC solutions. Finally, solutions were treated in the same manner as sample preparation.

#### UHPLC-MS/MS Conditions

The UHPLC-MS/MS analysis was performed on a Shimadzu LC-30AD system (Shimadzu Corporation, Kyoto, Japan) coupled with an Applied Biosystems Sciex 4,500 triple quadrupole MS/MS system (AB Sciex, Foster City, CA, United States). The separation was performed on an ACQUITY UPLC BEH C18 column (2.1 × 50 mm, 1.7 µm, Waters) with a Van Guard pre-column at 40°C. The mobile phase consisted of water (0.1% formic acid, v/v) as solvent A and acetonitrile (0.1% formic acid, v/v) as solvent B. The linear gradient elution steps were as follows: 0–0.5 min, 15–65% B; 0.5–1.4 min, 65% B; 1.4–1.41 min, 65–90% B; 1.41–1.80 min, 90% B; 1.80–1.81 min, 90–15% B; 1.81–2.3 min, 15% B. During 0–0.8 min, the flow was moved into waste. The flow rate and the autosampler temperature were set at 0.3 ml/min and 4°C, respectively. Biological samples were analysed in negative ion mode using an ESI source. The optimized multiple reaction monitoring (MRM) transitions were m/z 359.0–161.0 for RA, and m/z 481.0–301.0 for IS. Other relevant mass spectrometer parameters were as follows: ion spray voltage, −4500 V; source temperature, 550°C; curtain gas, 10 psi; ion source gas1, 55 psi; ion source gas2, 55 psi; collision gas, 8 psi; entrance potential, −10 V; collision cell exit potential, −11 V. Quantification of analytes was conducted using Analyst 1.6.3 and MultiQuant 3.0.2 softwares (AB Sciex, Foster City, CA, United States).

#### Method Validation

In order to prove the authenticity, reliability and reproducibility of the experimental data, the full bio-analytical method validation was carried out according to the US Food and Drug Administration (US-FDA) and European Medicines Agency guidance on bio-analytical method validation.

#### Data Analyses

Pharmacokinetic parameters of RA and its esters were calculated using the non-compartmental method of Phoenix WinNonlin 8.1 software (Pharsight, Mountain View, CA, United States). The absolute oral bioavailability was calculated using the following equation:

$$F\;\left(\%\right)=\frac{\left(AUC\;p.o.\;\times \;Dose\;i.v.\right)}{\left(AUC\;i.v.\;\times \;Dose\;p.o.\right)}\times \;100\%$$

### Biological Evaluation of Rosmarinic Acid Esters as Inhibitors of Vascular Smooth Muscle Cell Proliferation

#### Cell Culture

Primary VSMC were cultivated in DMEM-F12 (1:1) supplemented with 20% fetal bovine serum, 30 μg/ml gentamicin, and 15 ng/mL amphotericin B at 37°C in an incubator with 5% CO_2_ flow in a humidified atmosphere. Passages 4 to 12 were used in experiments.

#### Proliferation Assay

Resazurin conversion to fluorescent resorufin was used as a measure for cell proliferation. Primary VSMC were seeded at 5 × 10^3^ cells/well in a 96-well plate to grow for the next 24 h. Cells were then synchronized into G_0_ phase by serum deprivation for 24 h, pretreated with compounds for 30 min and stimulated with 20 ng/mL PDGF-BB for the next 48 h. After one washing step, cells were incubated in starvation medium containing 10 μg/mL resazurin for 2 h, and changes in fluorescence were monitored in a Tecan Spark (Tecan Group, Männedorf, Switzerland) plate reader at an excitation wavelength of 535 nm and an emission wavelength of 580 nm.

#### Cytotoxicity Assay

Increased lactate dehydrogenase (LDH) release is an indicator for the loss of cell membrane integrity, associated with cell death. Determination of LDH release in response to increasing concentrations of RAME (7), RAET (8), RABU (9), RAOCT (10) and RADOD (11) was performed as previously described (Liu et al., 2018).

#### Cell Cycle Analysis

Flow cytometry was used to analyze cell cycle progression. VSMC were seeded at a density of 10^5^ cells per well in 12-well plates. After rendered quiescent, cells were treated with increasing concentrations of RAME (**7**), RAET (**8**), RABU (**9**) and RAOCT (**10**) or with 0.1% DMSO for 30 min followed by stimulation with 20 ng/mL PDGF-BB for 16 h. Cells were collected by trypsinization, washed and resuspended in a hypotonic propidium iodide (PI) solution containing 0.1% (v/v) Triton X-100, 0.1% (w/v) sodium citrate, and 50 μg/mL PI. PI-stained nuclei were analyzed on a FACSCalibur (BD Biosciences, Vienna, Austria) flow cytometer at an excitation wavelength of 488 nm and an emission wavelength of 585 nm.

#### SDS-PAGE and Immunoblot Analysis

VSMC were seeded in 6-well plates at 2.5 × 10^5^ cells per well and cultivated for 24 h. Cells were serum-deprived for another 24 h, then pretreated for 30 min with RAET, RABU, RAOCT (all at 10 μmol/L) or vehicle (0.1% DMSO) and subsequently incubated with or without PDGF (20 ng/mL) for the indicated time. Afterward, cells were lysed with an ice-cold lysis buffer (50 mmol/L HEPES, 50 mmol/L NaCl, 10 mmol/L DTT, 50 mmol/L NaF, 10 mmol/L Na_4_P_2_O_7_ × 10 H_2_O, 5 mmol/L EDTA, 1 mmol/L Na_3_VO_4_), supplemented with 1 mmol/L PMSF, 1 × Complete™ (Roche Applied Science), and 1% (v/v) TritonX-100. Lysates were centrifuged at 5,600 × g at 4°C for 20 min, and supernatants were used for protein denaturation in 3 × SDS sample buffer for 8–10 min at 95°C. Protein concentrations were determined using Rotiquant reagent according to the manufacturer’s instructions (Carl Roth). Protein extracts (10 µg) were subjected to SDS-PAGE and immunoblot analysis. All antibodies were diluted as recommended by the providing company. Proteins were visualized using enhanced chemiluminescence reagent and quantified using a LAS-3000 luminescent image analyzer (Fujifilm) with AIDA software (Raytest).

#### Determination of Intracellular Bioavailability of RA Esters

After cells had been lysed and membranes had been removed by centrifugation, as described in the previous section, half of the cytoplasmic fractions were collected in 1.5 mL eppendorf tubes, diluted with methanol in 1:3 ratios and centrifuged at 5,600 × g for 20 min to remove proteins. Supernatants were then subjected to LC-MS/MS analysis to determine the cytoplasmic concentrations of RA esters at each treatment time point up to 16 h PDGF-BB stimulation. Cell culture media were also collected prior to the lysis procedure, diluted with methanol in a manner described above and subjected to LC-MS/MS. It was monitored whether the sum of cell lysate and culture medium concentrations of RA esters at the beginning of the experiment do not extensively deviate from treatment concentrations. Intracellular concentrations of RA esters were normalized to protein concentrations at each treatment time point. Details of the quantification method are shown in the Supplementary Material.

#### Statistical Analysis

Statistical analysis was performed using ANOVA/Bonferroni test. Data in the figures represent mean ± SD, and the number of experiments is given in the figure legends. All statistical analysis was performed using GraphPad PRISM 6 software, and a probability value <0.05 was considered significant.

## Results

### *In vivo* Bioavailability of Rosmarinic Acid and Its Esters

First, chemical stability and plasma stability of RA esters were assessed in solution (50% methanol with 0.1% formic acid) and in fresh rat plasma. The alkyl esters of RA remained stable during 5 h at room temperature in solution, while they converted to RA instantly in fresh plasma due to enzymatic hydrolysis (data not shown). As shown by others for ester-prodrugs of indomethacin and fusidic acid (Takahashi et al., 2018; Strydom et al., 2020), the alkyl esters of RA in our study were also hydrolyzed to form RA *in vivo*. Therefore, we developed and validated an UHPLC-MS/MS method to analyze the plasma concentration of RA after intravenous and oral administration of RA, RAME, RAET, RABU, RAOCT, RADOD in rats.

The method for determining RA in plasma was validated for selectivity, linearity, low limit of quantification (LLOQ), precision, accuracy, recovery, matrix effect, and stability. Details of the method validation are presented in the Supplementary Material. Typical chromatograms are shown in Supplementary Figure S8, with no endogenous interfering peaks in the chromatograms of blank plasma at retention times of RA and IS. The method displayed good linearity in the range of 1–2000 ng/mL with the correlation coefficient greater than 0.999. The LLOQ was 1 ng/mL with the signal-to-noise ratio of ˃10:1. Precision, accuracy, recovery and matrix effect of RA in rat plasma are shown in Supplementary Table S1. Stability of RA under different storage conditions is shown in Supplementary Table S2.

**FIGURE 3:**
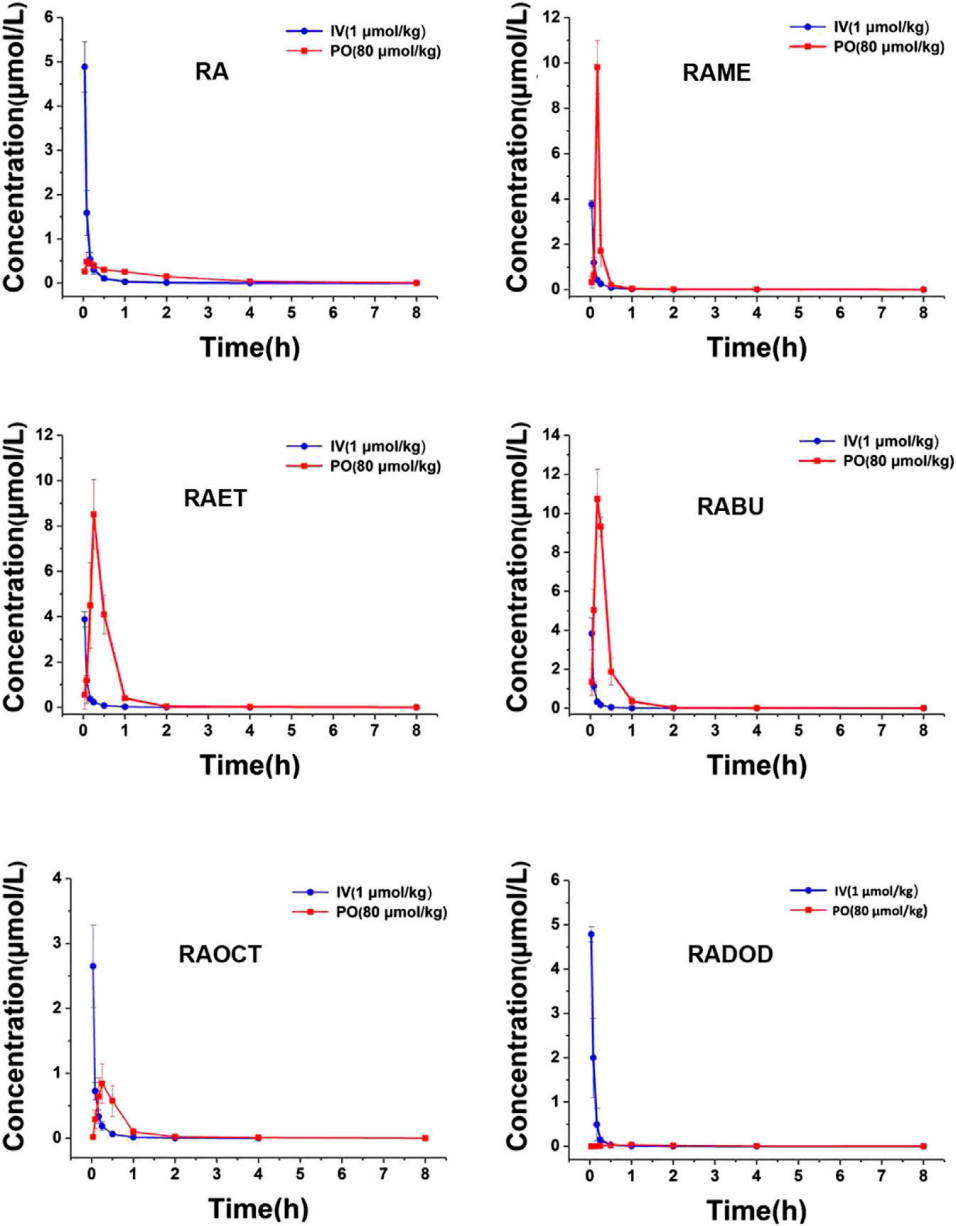
Mean rat plasma concentration-time curves of RA after intravenous (1 μmol/kg) and oral (80 μmol/kg) administration of RA, RAME, RAET, RABU, RAOCT, RADOD, respectively. Data are presented as mean ± SD, *n* = 3.

The plasma concentrations of RA after the intravenous (1 μmol/kg) and oral (80 μmol/kg) administrations of RA and its esters were determined by the validated LC-MS/MS method. All mean plasma concentration-time profiles are shown in Figure 3. The main pharmacokinetic parameters including area under the curve (AUC), maximum plasma concentration (C_max_), time to reach C_max_ (T_max_), plasma half-life (T_1/2_), apparent volume of distribution (V_d_), clearance (CL), and bioavailability (F) are shown in Table 1. After oral administration of RA (**6**), RAME (**7**), RAET (**8**), RABU (**9**), RAOCT (**10**), RADOD (**11**) at 80 μmol/kg, the C_max_ of RA were 0.55 ± 0.16, 9.81 ± 1.18, 8.51 ± 1.53, 10.98 ± 1.13, 0.84 ± 0.30 and 0.04 ± 0.01 μmol/L, respectively. The absolute bioavailability of RA after RA, RAME, RAET, RABU, RAOCT and RADOD administration were 1.57%, 3.30%, 9.65%, 10.52%, 1.93% and 0.22%, respectively. With the increase of the alkyl chain length, T_max_ and T_1/2_ did not show obvious changes, while the C_max_ and bioavailability values increased only up to C4 alkyl chain. When applied in the form of the butyl-ester (RABU), the C_max_ and the bioavailability of RA increased around 20- and 7-fold, respectively.

**TABLE 1:** Pharmacokinetic parameters of RA after intravenous (1 μmol/kg) and oral (80 μmol/kg) administration of RA or its alkyl esters.

Parameters^a^		AUC_0-t_ (h*µmol/L)	AUC_0-∞_ (h*µmol/L)	C_max_ (µM)	T_max_ (h)	T_1/2_ (h)	V_d_ (L/kg)	CL (L/h/kg)	F (%)
RA (6)	iv	0.64 ± 0.09	0.64 ± 0.09	–^b^	–	0.22 ± 0.07	0.49 ± 0.08	1.58 ± 0.21	–
	po	0.78 ± 0.09	0.80 ± 0.07	0.55 ± 0.16	0.17 ± 0.08	1.31 ± 0.23			1.57
RAME (7)	iv	0.50 ± 0.04	0.50 ± 0.04	–	–	0.33 ± 0.01	0.97 ± 0.09	2.01 ± 0.15	–
	po	1.30 ± 0.15	1.32 ± 0.15	9.81 ± 1.18	0.17 ± 0	1.04 ± 0.11			3.30
RAET (8)	iv	0.49 ± 0.01	0.49 ± 0.01	–	–	0.30 ± 0.08	0.87 ± 0.25	2.02 ± 0.03	–
	po	3.81 ± 0.36	3.82 ± 0.37	8.51 ± 1.53	0.25 ± 0	0.51 ± 0.09			9.65
RABU (9)	iv	0.46 ± 0.10	0.46 ± 0.10	–	–	0.25 ± 0.07	0.80 ± 0.16	2.27 ± 0.58	–
	po	3.86 ± 0.28	3.86 ± 0.27	10.98 ± 1.13	0.19 ± 0.05	0.53 ± 0.10			10.52
RAOCT (10)	iv	0.36 ± 0.09	0.36 ± 0.09	–	–	0.49 ± 0.06	1.99 ± 0.32	2.91 ± 0.87	–
	po	0.55 ± 0.20	0.56 ± 0.19	0.84 ± 0.30	0.25 ± 0	0.62 ± 0.15			1.93
RADOD (11)	iv	0.56 ± 0.07	0.57 ± 0.07	–	–	0.22 ± 0.02	0.56 ± 0.09	1.79 ± 0.22	–
	po	0.09 ± 0.02	0.10 ± 0.02	0.04 ± 0.01	1.00 ± 0	1.78 ± 0.20			0.22

Data are presented as mean ± SD.

^a^ AUC0-t, area under the curve from zero to the last measurable time; AUC0-∞, area under the curve from zero to time infinity; Cmax, maximum plasma concentration; Tmax, time to reach Cmax; T1/2, plasma half-life; Vd, apparent volume of distribution; CL, clearance; F, bioavailability.

^b^ not available.

### Rosmarinic Acid Esters, Not Metabolites, Suppress the PDGF-Induced Vascular Smooth Muscle Cell Proliferation

**FIGURE 4:**
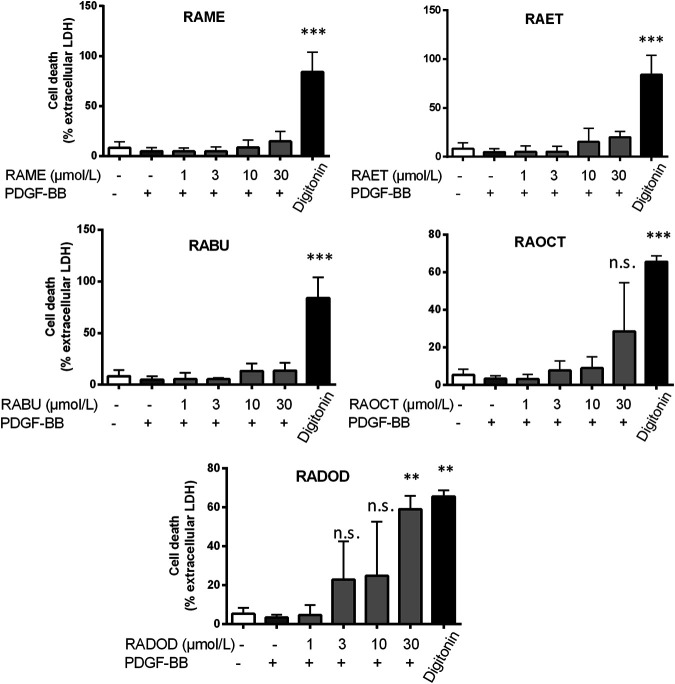
Cytotoxicity of RA esters in PDGF-induced VSMC. VSMC were pretreated with 0.1% DMSO or increasing concentrations of RA esters as indicated for 30 min, stimulated with 20 ng/mL PDGF-BB for 24 h, and released LDH was determined. Digitonin (80 μmol/L) was used as a positive control. Graphs show means ± SD out of 3 (RAME, RAET and RABU) and 4 (RAOCT and RADOD) independent experiments (n.s., not significant, *p* > 0.05, **p < 0.01, ***p < 0.001, ANOVA/Bonferroni, “vehicle with PDGF-BB” vs. “RA ester with PDGF-BB”).

The antiproliferative effect of RA and, in particular, of its methyl ester (RAME) in VSMC was described in an earlier study (Liu et al., 2018). We, therefore, decided to examine: 1) whether an increase in the length of an alkyl chain could enhance the potency of RA esters in VSMC and 2) whether other known RA metabolites (Baba et al., 2005) (Figure 1) might have an effect on VSMC proliferation as well. RA (6) and its esters RAME (7), RAET (8), RABU (9), RAOCT (10) and RADOD (11), as well as RA metabolites: 2, 3, 4 and 5 were all tested at 10 μmol/L in PDGF-BB-activated VSMC using the resazurin assay. RA metabolites, including 1 at 50 μmol/L, did not render active at tested concentrations, whereas all the tested esters of RA completely blocked the PDGF-triggered increase of VSMC metabolic activity, as surrogate indicator for proliferation (Table 2). A consistent reduction in VSMC biomass by the RA esters was observed in the crystal violet assay (data not shown). Determining the IC_50_ values of RA and of each of the tested RA esters revealed a decrease in IC_50_ from 7.9 μmol/L in RAME-treated to 3.37 μmol/L in RAET-treated VSMC. A further slight decrease in IC_50_ was observed by extending the alkyl chain of the ester group up to C8-ester (Table 2). In the case of the RADOD, VSMC proliferation was unaffected at 1 μmol/L, but completely abrogated at 3 μmol/L of concentration, which indicated a possible toxic effect of this compound in VSMC (data not shown). Therefore, we examined the LDH release in VSMC treated with all five RA esters. RAME (7), RAET (8) and RABU (9) did not induce cell death up to 30 μmol/L, whereas RAOCT (10)-treated VSMC showed a marked but not significant increase in LDH production at 30 μmol/L (Figure 4). RADOD (11)-induced cell death at 30 μmol/L was comparable to that of the positive control, digitonin. At lower concentrations, 3 and 10 μmol/L, RAOCT also induced a distinct LDH release, which however did not reach significance (Figure 4). Due to the exhibited cytotoxic effect, RADOD (11) was excluded from further *in vitro* experiments.

**TABLE 2:** Antiproliferative effects of RA esters and metabolites on VSMCs quantified by the resazurin conversion assay.

Compound	VSMC proliferation at 10 μmol/L (relative to vehicle control, RU)	IC50 (µmol/L)
RA (6)	0.781 ± 0.167^n.s.^	n.d.
RAME (7)	0.415 ± 0.109^a^	7.90
RAET (8)	0.328 ± 0.081^a^	3.37
RABU (9)	0.308 ± 0.052^a^	2.84
RAOCT (10)	0.338 ± 0.147^a^	2.22
RADOD (11)	0.054 ± 0.081^a^	n.d.
1 (50 μmol/L)	0.922 ± 0.124^n.s.^	–
2	0.940 ± 0.220^n.s.^	–
3	1.072 ± 0.152^n.s.^	–
4	1.068 ± 0.173^n.s.^	–
5	1.205 ± 0.278^n.s.^	–

Results are presented as mean ± SD, relative to the PDGF-BB-stimulated vehicle control, and IC_50_ values were determined if the compound (10 μmol/L) showed a relative inhibitory action lower than 0.75 RU. All values were obtained from a minimum of three independent experiments.

^a^ *p* < 0.001; n.s. not significant compared to vehicle control.

–not tested; n.d.: unable to calculate.

### Ethyl-, Butyl and Octyl Esters Have the Same Mode of Action as the Methyl Ester of Rosmarinic Acid in Vascular Smooth Muscle Cells

**FIGURE 5:**
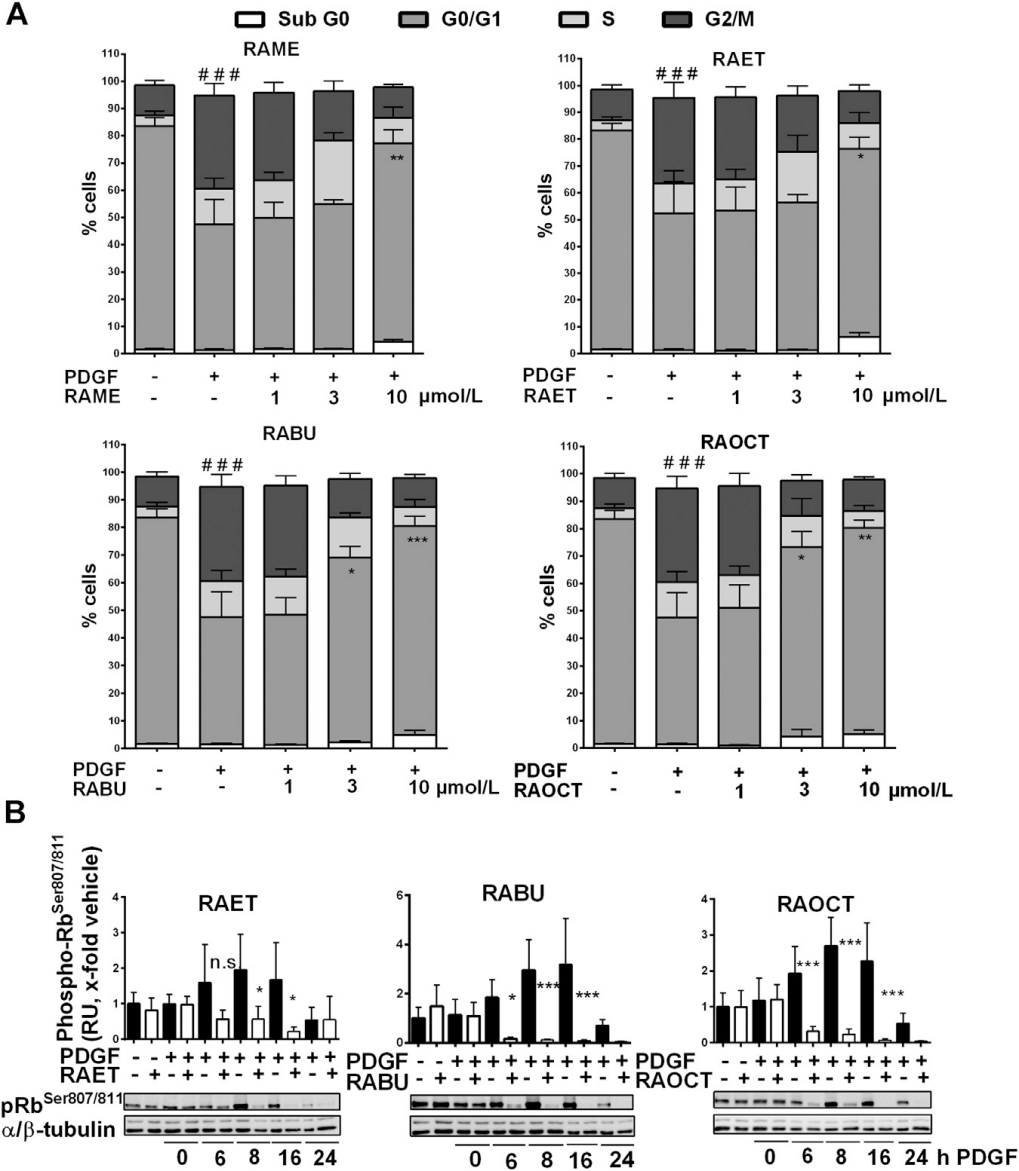
Ethyl, butyl and octyl esters of rosmarinic acid (RA) inhibit the proliferation of VSMC by exerting the same mechanism of action as the methyl ester **(A)** Quiescent VSMC were treated with 0.1% DMSO or increasing concentrations of RA esters as indicated for 30 min, stimulated with 20 ng/mL PDGF-BB for 16 h and the cell cycle progression was analyzed using PI staining and flow cytometry. Graphs show means ± SD out of three independent experiments (G_2_/M phase: ###*p* < 0.001, ANOVA/Bonferroni, “vehicle” vs. “vehicle with PDGF-BB”; G_0_/G_1_ phase: *p < 0.05, **p < 0.01, ***p < 0.001, ANOVA/Bonferroni, “vehicle with PDGF-BB” vs. “compound with PDGF-BB”) **(B)** Quiescent VSMC were treated with 10 μmol/L of indicated RA esters or 0.1% DMSO for 30 min and stimulated with 20 ng/mL PDGF-BB for indicated amounts of time. Lyzed cells were subjected to western blot analysis for phospho-Rb^Ser807/811^ detection. α/β-tubulin was used as loading control. Representative blots together with compiled results of densitometric analyses out of four independent experiments are shown (mean ± SD, n.s. not significant, *p < 0.05, **p < 0.01, ***p < 0.001, ANOVA/Bonferroni, “vehicle” vs. “RA ester”).

We reported previously that RAME induces a G_0_/G_1_ cell cycle arrest in PDGF-induced VSMC and suppresses retinoblastoma protein (Rb) phosphorylation, presumably by inhibiting the activity of the cyclin-dependent kinase 2 (CDK2) (Liu et al., 2018). To examine in what manner esters of RA with longer alkyl chains affect cell cycle progression, we subjected the PDGF-BB-induced VSMC treated with increasing concentrations of RAME (**7**), RAET (**8**), RABU (**9**) and RAOCT (**10**) to PI staining and flow cytometry analysis. While control VSMC markedly progressed into G_2_/M phase of the cell cycle after 16 h PDGF stimulation, all tested RA esters applied at 10 μmol/L arrested VSMC in G_0_/G_1_ phase (Supplementary Figure S9 and Figure 5A). Furthermore, treatment with RABU and RAOCT at 3 μmol/L resulted in a significantly higher percentage of cells in G_0_/G_1_ compared to vehicle control (Figure 5A). Rb protein phosphorylation at Ser^807/811^ primes the protein for further phosphorylation steps, which are a prerequisite for S-phase entry (Rubin, 2013). To examine whether the esters of RA affect the phosphorylation at Ser^807/811^, we performed a time-course experiment with ethyl-, butyl- and octyl ester in PDGF-BB-stimulated VSMC and a subsequent western blot analysis. All of the three tested RA esters prevented Rb protein phosphorylation at Ser^807/811^ that had been markedly increased after 8 and 16 h of PDGF-BB stimulation (Figure 5B). These data strongly indicate that ethyl-, butyl and octyl esters exhibit the same mode of action as previously reported for the methyl ester of RA (Liu et al., 2018).

### Rosmarinic Acid Esters Are Absorbed in Cultured Vascular Smooth Muscle Cells in Their Original Esterified Form

**FIGURE 6:**
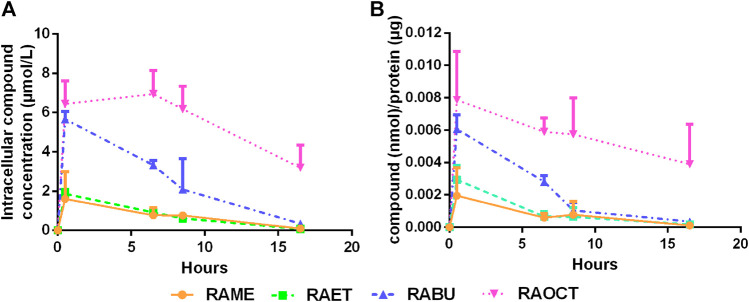
Esters of rosmarinic acid with longer alkyl groups show increased intracellular bioavailability. Cytoplasmic fractions of cells treated with rosmarinic acid (RA) esters (10 μmol/L) for up to 16 h were diluted with MeOH, centrifuged to remove proteins and subjected to LC-MS/MS analysis **(A)** Graph shows means ± SD of detected concentrations of RA esters within cytoplasmic fractions over time out of three independent experiments **(B)** Detected concentrations of respective esters were normalized to protein concentrations. Graph shows mean ± SD of the amount of compound per µg protein out of three independent experiments.

Next, we examined whether RA esters exert their intracellular effects as esters or whether they become hydrolyzed into RA. This was achieved by analyzing the contents of RA and its esters in cytoplasmic fractions of VSMC treated with 10 μmol/L concentration of RAME (**7**), RAET (**8**), RABU (**9**) and RAOCT (**10**) for 0.5, 6.5, 8.5 and 16.5 h by LC-MS/MS. Since only the respective RA esters but no free RA could be successfully identified and quantified both in cell lysates and in culture media supernatants at any treatment time point, we conclude that the active principle of RA esters in an *in vitro* setting of cultured VSMC lies in their esterified form. Intracellularly accumulated concentrations after 0.5 h exposure to 10 μmol/L of RABU and of RAOCT were about 6 μmol/L, and 3-fold higher than those of RAME and RAET (Figure 6A). We then normalized the detected cytoplasmic concentration of each RA ester to the protein content of the respective cell lysate to account for possible changes in cell number (Figure 6B). This normalization step did not tremendously affect the trend of intracellular RA ester bioavailability over time. However, it produced a more obvious correlation between increased alkyl chain length and initial intracellular bioavailability (Figure 6B). The intracellular contents of RAME, RAET and RABU gradually decreased over time, reaching around 2 pmol per µg of protein at 8.5 h. The concentration of RAOCT within VSMC also slightly declined over time but remained relatively high, even at the 16.5 h time point (Figures 6A,B). The inability of VSMC to efficiently eliminate RAOCT points to an increase in intracellular accumulation of this compound when administered at concentrations higher than 10 μmol/L. This, in turn, might lead to cytotoxicity, as evident by a slight increase in LDH release upon treatment with this compound at 30 μmol/L (Figure 4). Our results strongly indicate that, of all tested RA esters, RABU possesses the best intracellular bioavailability.

## Discussion

RA esters, especially RAME, widely exist in medicinal plants (e.g. thyme, garden and red sage), but in much lower amounts compared to RA (Li et al., 1993; Fecka and Turek, 2008; Putnik et al., 2016). In recent years, both plant-derived and synthetic RA esters have been studied and showed potent biological activities (Abedini et al., 2013; Kang et al., 2016; Thammason et al., 2018). Out of four isolated active compounds from the hydromethanolic stem extract of *Hyptis atrorubens* (*Lamiaceae*), RAME showed the most potent antimicrobial activity (Abedini et al., 2013). Synthetic RAET acted vasorelaxant in rat aorta (Wicha et al., 2015) and anti-inflammatory in LPS-induced MH-S cells (Thammason et al., 2018). RABU was isolated from *Isodon oresbius* in 1999 (Huang et al., 1999), and was found cytotoxic against colon, ovary and melanin cancer cell lines (Huang et al., 2006). It was reported that antioxidant, anti-allergic, and antimicrobial activities of RA esters tend to be parabolic with the ester chain increasing (Laguerre et al., 2010; Suriyarak et al., 2013; Zhu et al., 2015). However, to the best of our knowledge, there are no studies examining the oral bioavailability of RA alkyl esters or their effect on VSMC proliferation.

In this study we explored the possibility of improving the bioavailability of RA and its biological activity in proliferating VSMC by esterification with alkyl chains of increasing length. RA esters are rapidly hydrolyzed in fresh rat plasma into RA and the corresponding alcohols. Esterification of RA with up to 4C-alkyl chains increased the *in vivo* bioavailable fraction of RA from 2 to 7-fold, whereas further increase in alkyl chain length resulted in low bioavailability of RA. Regarding the antiproliferative effect in VSMC, derivatization of RA with methyl-, ethyl-, butyl- and octyl-esters resulted in a decrease of the IC_50_ values, whereas RA dodecyl ester was found to be cytotoxic. Contrary to the observed hydrolysis in blood plasma, RA esters were detected in cells in their original form. Treatment with butyl- and octyl-esters achieved the highest intracellular concentrations.

Caffeic (**1**), 3,4-dihydroxyphenyl lactic (**2**), *m*-coumaric (**3**), ferulic (**4**) and *m*-hydroxyphenylpropionic (**5**) acids were found to account for the bioavailable fraction of ingested RA to a larger extent than RA itself (Nakazawa and Ohsawa, 1998). The biological activity of certain polyphenols could in fact be attributed to their bioavailable metabolites, as exemplified by the well-known metabolite of the isoflavone daidzein, S (-)-equol (Setchell and Clerici, 2010). However, in the case of the antiproliferative effect of RA in VSMC, we show that the active principle is RA, rather than any of the so far reported gut-microbial metabolites of RA.

Therefore, we concentrated on improving the absolute bioavailability and the potency of RA to inhibit VSMC proliferation by increasing its lipophilicity via esterification. After oral/intravenous administration of RA alkyl esters to rats, the bioavailability of RA does not entirely progress with the increase of the alkyl ester chain length from methyl to dodecyl. From methyl to butyl side chains, the bioavailability gradually increases. From butyl to dodecyl chains, the bioavailability decreases. Oral administration of RA ethyl- and butyl-esters in rats increased the bioavailability of RA up to around 7-fold, and the respective C_max_ values were in the range of previously reported IC_50_ values of RA against the PDGF-BB-induced VSMC proliferation (Liu et al., 2018). Our *in vivo* pharmacokinetic results showing T_max_ at around 0.2 h (apart from RADOD) indicate that RA, as well as RA esters are being absorbed in the upper parts of the GIT and are eliminated fast. Bioavailability and T_max_ data for RA presented here are well in accordance with previous studies performed in rats (Nakazawa and Ohsawa, 1998; Konishi et al., 2005; Wang et al., 2017). Previous reports on RA absorption suggest the involvement of paracellular diffusion of RA in the upper intestine (Konishi et al., 2005; Konishi and Kobayashi, 2005), in contrast to the caffeic acid that is being absorbed via monocarboxylic transporters (MCT), and showed about 10 times higher absorption efficiency (Konishi et al., 2005). It would, thus, be interesting to examine whether esterification of RA, with ethanol and butanol in particular, increases its diffusion properties or whether some cellular transporters are involved. Unlike methyl to octyl esters of RA, oral administration of dodecyl ester decreased the bioavailability of RA. A possible reason for this might be the poor dissolution of RADOD in the aqueous milieu of the gastrointestinal tract, which is almost always a prerequisite for oral absorption.

In contrast to systemic application, in cultured VSMC, RA esters did not undergo hydrolysis by intracellular esterases and remained in their original form. This result calls for caution when interpreting results of ester prodrugs in cultured cells, as these cells might lack the necessary carboxylesterases. In the context of local drug delivery to prevent aberrant VSMC proliferation and neointima formation, as in the case of antiproliferative drugs used in drug-eluting stents (Alfonso et al., 2014), RABU achieved the highest intracellular concentration (60% of applied concentration) and the highest potency against VSMC proliferation, without exhibiting significant toxicity.

We showed previously that RAME inhibits VSMC proliferation by decreasing the Rb protein phosphorylation, presumably via direct inhibition of CDK2, and subsequently arresting cells in G_0_/G_1_ phase (Liu et al., 2018). Our current results strongly indicate that the esterification of RA with alkyl chains longer than one carbon atom does not alter the mechanism of action. While RAME and RAET were detected intracellularly at only about 20% of the administered concentration, levels of absorbed RABU and RAOCT were about 3- and 4-fold higher, respectively. This was reflected in the cell-cycle progression results, as RABU and RAOCT arrested VSMC in G_0_/G_1_ phase at lower applied concentrations than RAME and RAET. The inhibition of Rb protein phosphorylation was also more pronounced after RABU and RAOCT administration.

Intracellular concentrations of RA esters increased shortly after the treatment, as detected at the 30 min time point. RA esters-mediated inhibition of PDGF-induced Rb protein phosphorylation was, however, observed 6 h after RAME, RAET and RABU had intracellularly reached their peak concentrations. In our previous study, RAME could inhibit the CDK2 kinase activity *in vitro* with an IC_50_ of 11.4 μmol/L, while the activity of CDK4 was affected only at much higher concentrations (Liu et al., 2018). The expression and activity of CDK2 becomes relevant at later stages of the G_1_ phase and at the transition into S-phase of the cell cycle (Tadesse et al., 2019). CDK3/cyclin C complex was shown to be crucial for the transition of dormant G_0_ cells into the G_1_ phase, by phosphorylating the Rb protein at Ser^807/811^ (Ren and Rollins, 2004). CDK2 and CDK3 share 87% alignment score in their ATP binding pocket regions (Sridhar et al., 2006). It would, thus, be quite interesting to examine whether RA esters act already at the PDGF-induced cell cycle entry by inhibiting the activity of CDK3.

In summary, this study is the first to investigate how derivatization of rosmarinic acid affects both its bioavailability and biological activity. Of all tested esters, esterification of RA with a C4-alkyl chain resulted in the highest bioavailable fraction of RA *in vivo* and the highest potency against VSMC proliferation *in vitro*. Considering the enhanced intracellular bioavailability, RABU might exhibit an even more potent effect against neointima formation in a femoral artery cuff model than previously shown for RAME (Liu et al., 2018). However, further studies are needed to investigate the safety of long-term RABU use as well as its effect on the neointima formation *in vivo*.

## Data Availability Statement

The raw data supporting the conclusions of this article will be made available by the authors, without undue reservation.

## Ethics Statement

The animal study was reviewed and approved by Ethics Committee of Yantai University (IACUC No. 2018-DA-12).

## Author Contributions

TB and RL designed the study and drafted the manuscript; TB, GR, PH, LD, and GY performed experiments and analyzed data; EH and VD supervised the execution of experiments and their interpretation. All authors revised and approved the final manuscript.

## Funding

This work is supported by the National Natural Science Foundation of China (grant numbers 81973513, and 81603326). Open Access Publication fee was funded by the University of Vienna.

## Conflict of Interest

The authors declare that the research was conducted in the absence of any commercial or financial relationships that could be construed as a potential conflict of interest.

## Abbreviations:

AUC, area under the curve; Cmax, maximum plasma concentration, CL, clearance; CDK, cyclin-dependent kinase; DMAP, 4-dimethylamino-pyridine; EDC, 1-(3-dimethylaminopropyl)-3-ethylcarbodiimide; F, bioavailability; IS, internal standard; LDH, lactate dehydrogenase; MRM, multiple reaction monitoring; PDGF, platelet-derived growth factor; PI, propidium iodide; VSMC, vascular smooth muscle cell; RA, rosmarinic acid; RABU, rosmarinic acid butyl ester; RAET, rosmarinic acid ethyl ester; RAME, rosmarinic acid methyl ester; RADOD, rosmarinic acid dodecyl ester; RAOCT, rosmarinic acid octyl ester; Rb, retinoblastoma; T_max_, time to reach C_max;_ T_1/2_, plasma half-life; V_d_, apparent volume of distribution.

## References

[B1] Abedini A. Roumy V. Mahieux S. Biabiany M. Standaert-Vitse A. Rivière C. (2013). Rosmarinic acid and its methyl ester as antimicrobial components of the hydromethanolic extract of *Hyptis atrorubens* poit. (Lamiaceae). Evid. Based Complem. Alternat. Med. 2013, 604536 10.1155/2013/604536 PMC3855952 24348709

[B2] Alfonso F. Byrne R. A. Rivero F. Kastrati A. (2014). Current treatment of in-stent restenosis. J. Am. Coll. Cardiol. 63, 2659–2673. 10.1016/j.jacc.2014.02.545 24632282

[B3] Baba S. Osakabe N. Natsume M. Yasuda A. Muto Y. Hiyoshi K. (2005). Absorption, metabolism, degradation and urinary excretion of rosmarinic acid after intake of Perilla frutescens extract in humans. Eur. J. Nutr. 44, 1–9. 10.1007/s00394-004-0482-2 15309457

[B4] Bel-Rhlid R. Crespy V. Pagé-Zoerkler N. Nagy K. Raab T. Hansen C. E. (2009). Hydrolysis of rosmarinic acid from rosemary extract with esterases and Lactobacillus johnsonii *in vitro* and in a gastrointestinal model. J. Agric. Food Chem. 57, 7700–7705. 10.1021/jf9014262 19658402

[B5] Biasutto L. Marotta E. De Marchi U. Zoratti M. Paradisi C. (2007). Ester-based precursors to increase the bioavailability of quercetin. J. Med. Chem. 50, 241–253. 10.1021/jm060912x 17228866

[B6] Fecka I. Turek S. (2008). Determination of polyphenolic compounds in commercial herbal drugs and spices from Lamiaceae: thyme, wild thyme and sweet marjoram by chromatographic techniques. Food Chem. 108, 1039–1053. 10.1016/j.foodchem.2007.11.035 26065769

[B7] Hamada N. M. Abdo N. Y. (2015). Synthesis, characterization, antimicrobial screening and free-radical scavenging activity of some novel substituted pyrazoles. Molecules 20, 10468–10486. 10.3390/molecules200610468 26060913 PMC6272688

[B8] Hu J. N. Zou X. G. He Y. Chen F. Deng Z. Y. (2016). Esterification of quercetin increases its transport across human Caco-2 cells. J. Food Sci. 81, H1825–H1832. 10.1111/1750-3841.13366 27301074

[B9] Huang H. Chao Q. R. Tan R. X. Sun H. D. Wang D. C. Ma J. (1999). A new rosmarinic acid derivative from Isodon oresbius. Planta Med. 65, 92–93. 10.1055/s-2006-960451 17260244

[B10] Huang L. J. Li C. H. Lu Z. M. Ma Z. B. Yu D. Q. (2006). Total synthesis and biological evaluation of (+)- and (-)-Butyl ester of rosmarinic acid. J. Asian Nat. Prod. Res. 8, 561–566. 10.1080/10286020500176229 16931433

[B11] Kang J. Tang Y. Liu Q. Guo N. Zhang J. Xiao Z. (2016). Isolation, modification, and aldose reductase inhibitory activity of rosmarinic acid derivatives from the roots of Salvia grandifolia. Fitoterapia 112, 197–204. 10.1016/j.fitote.2016.05.011 27233987

[B12] Konishi Y. Hitomi Y. Yoshida M. Yoshioka E. (2005). Pharmacokinetic study of caffeic and rosmarinic acids in rats after oral administration. J. Agric. Food Chem. 53, 4740–4746. 10.1021/jf0478307 15941309

[B13] Konishi Y. Kobayashi S. (2005). Transepithelial transport of rosmarinic acid in intestinal Caco-2 cell monolayers. Biosci. Biotechnol. Biochem. 69, 583–591. 10.1271/bbb.69.583 15784988

[B14] Laguerre M. López Giraldo L. J. Lecomte J. Figueroa-Espinoza M. C. Baréa B. Weiss J. (2010). Relationship between hydrophobicity and antioxidant ability of "phenolipids" in emulsion: a parabolic effect of the chain length of rosmarinate esters. J. Agric. Food Chem. 58, 2869–2876. 10.1021/jf904119v 20131842

[B15] Levitzki A. (2004). PDGF receptor kinase inhibitors for the treatment of PDGF driven diseases. Cytokine Growth Factor Rev. 15, 229–235. 10.1016/j.cytogfr.2004.03.010 15207814

[B16] Li J. He L. Y. Song W. Z. (1993). Separation and quantitative determination of seven aqueous depsides in Salvia miltiorrhiza by HPTLC scanning. Yao Xue Xue Bao 28, 543–547. 8285058

[B17] Liu R. Heiss E. H. Waltenberger B. Blažević T. Schachner D. Jiang B. (2018). Constituents of Mediterranean spices counteracting vascular smooth muscle cell proliferation: identification and characterization of rosmarinic acid methyl ester as a novel inhibitor. Mol. Nutr. Food Res. 62, e1700860 10.1002/mnfr.201700860 29405576

[B18] Mosele J. I. Martín-Peláez S. Macià A. Farràs M. Valls R. M. Catalán Ú. (2014). Study of the catabolism of thyme phenols combining *in vitro* fermentation and human intervention. J. Agric. Food Chem. 62, 10954–10961. 10.1021/jf503748y 25339317

[B19] Nakamura Y. Ohto Y. Murakami A. Ohigashi H. (1998). Superoxide scavenging activity of rosmarinic acid from *Perilla frutescens* Britton var. acuta f. viridis. J. Agric. Food Chem. 46, 4545–4550. 10.1021/jf980557m

[B20] Nakazawa T. Ohsawa K. (1998). Metabolism of rosmarinic acid in rats. J. Nat. Prod. 61, 993–996. 10.1021/np980072s 9722482

[B21] Noguchi-Shinohara M. Ono K. Hamaguchi T. Iwasa K. Nagai T. Kobayashi S. (2015). Pharmacokinetics, safety and tolerability of Melissa officinalis extract which contained rosmarinic acid in healthy individuals: a randomized controlled trial. PloS One 10, e0126422 10.1371/journal.pone.0126422 25978046 PMC4433273

[B22] Nunes S. Madureira A. R. Campos D. Sarmento B. Gomes A. M. Pintado M. (2017). Therapeutic and nutraceutical potential of rosmarinic acid-Cytoprotective properties and pharmacokinetic profile. Crit. Rev. Food Sci. Nutr. 57, 1799–1806. 10.1080/10408398.2015.1006768 26114303

[B23] Petersen M. (2013). Rosmarinic acid: new aspects. Phytochem. Rev. 12, 207–227. 10.1007/s11101-013-9282-8

[B24] Putnik P. Kovačević D. B. Penić M. Fegeš M. Dragović-Uzelac V. (2016). Microwave-assisted extraction (MAE) of dalmatian sage leaves for the optimal yield of polyphenols: HPLC-DAD identification and quantification. Food Anal. Methods 9, 2385–2394. 10.1007/s12161-016-0428-3

[B25] Qiang Z. Ye Z. Hauck C. Murphy P. A. Mccoy J. A. Widrlechner M. P. (2011). Permeability of rosmarinic acid in Prunella vulgaris and ursolic acid in Salvia officinalis extracts across Caco-2 cell monolayers. J. Ethnopharmacol. 137, 1107–1112. 10.1016/j.jep.2011.07.037 21798330 PMC3202029

[B26] Ren S. Rollins B. J. (2004). Cyclin C/cdk3 promotes Rb-dependent G0 exit. Cell 117, 239–251. 10.1016/s0092-8674(04)00300-9 15084261

[B27] Rubin S. M. (2013). Deciphering the retinoblastoma protein phosphorylation code. Trends Biochem. Sci. 38, 12–19. 10.1016/j.tibs.2012.10.007 23218751 PMC3529988

[B28] Setchell K. D. Clerici C. (2010). Equol: pharmacokinetics and biological actions. J. Nutr. 140, 1363S–8S. 10.3945/jn.109.119784 20519411 PMC2884334

[B29] Sridhar J. Akula N. Pattabiraman N. (2006). Selectivity and potency of cyclin-dependent kinase inhibitors. AAPS J. 8, E204–E221. 10.1208/aapsj080125 16584130 PMC2751441

[B30] Strydom N. Kaur G. Dziwornu G. A. Okombo J. Wiesner L. Chibale K. (2020). Pharmacokinetics and organ distribution of C-3 alkyl esters as potential antimycobacterial prodrugs of fusidic acid. ACS Infect. Dis. 6, 459–466. 10.1021/acsinfecdis.9b00405 32011859

[B31] Suriyarak S. Bayrasy C. Schmidt H. Villeneuve P. Weiss J. (2013). Impact of fatty acid chain length of rosmarinate esters on their antimicrobial activity against Staphylococcus carnosus LTH1502 and *Escherichia coli* K-12 LTH4263. J. Food Protect 76, 1539–1548. 10.4315/0362-028X.JFP-12-254 23992498

[B32] Tadesse S. Caldon E. C. Tilley W. Wang S. (2019). Cyclin-dependent kinase 2 inhibitors in cancer therapy: an update. J. Med. Chem. 62, 4233–4251. 10.1021/acs.jmedchem.8b01469 30543440

[B33] Takahashi M. Ogawa T. Kashiwagi H. Fukushima F. Yoshitsugu M. Haba M. (2018). Chemical synthesis of an indomethacin ester prodrug and its metabolic activation by human carboxylesterase 1. Bioorg. Med. Chem. Lett. 28, 997–1000. 10.1016/j.bmcl.2018.02.035 29503023

[B34] Thammason H. Khetkam P. Pabuprapap W. Suksamrarn A. Kunthalert D. (2018). Ethyl rosmarinate inhibits lipopolysaccharide-induced nitric oxide and prostaglandin E2 production in alveolar macrophages. Eur. J. Pharmacol. 824, 17–23. 10.1016/j.ejphar.2018.01.042 29391157

[B35] Villalva M. Jaime L. Aguado E. Nieto J. A. Reglero G. Santoyo S. (2018). Anti-inflammatory and antioxidant activities from the basolateral fraction of Caco-2 cells exposed to a rosmarinic acid enriched extract. J. Agric. Food Chem. 66, 1167–1174. 10.1021/acs.jafc.7b06008 29345918

[B36] Wang J. X. Li G. Y. Rui T. Q. Kang A. Li G. C. Fu T. M. (2017). Pharmacokinetics of rosmarinic acid in rats by LC-MS/MS: absolute bioavailability and dose proportionality. RSC Adv. 7, 9057–9063. 10.1039/C6RA28237G

[B37] Wicha P. Tocharus J. Nakaew A. Pantan R. Suksamrarn A. Tocharus C. (2015). Ethyl rosmarinate relaxes rat aorta by an endothelium-independent pathway. Eur. J. Pharmacol. 766, 9–15. 10.1016/j.ejphar.2015.09.003 26362751

[B38] Zhu F. Xu Z. Yonekura L. Yang R. Tamura H. (2015). Antiallergic activity of rosmarinic acid esters is modulated by hydrophobicity, and bulkiness of alkyl side chain. Biosci. Biotechnol. Biochem. 79, 1178–1182. 10.1080/09168451.2015.1010478 25686361

[B39] Zoric Z. Markic J. Pedisic S. Bucevic-Popovic V. Generalic-Mekinic I. Grebenar K. (2016). Stability of rosmarinic acid in aqueous extracts from different Lamiaceae species after *in vitro* digestion with human gastrointestinal enzymes. Food Technol. Biotechnol. 54, 97–102. 10.17113/ftb.54.01.16.4033 27904398 PMC5105624

