# Supplementary material for: Short Chain (≤C4) Esterification Increases Bioavailability of Rosmarinic Acid and Its Potency to Inhibit Vascular Smooth Muscle Cell Proliferation
Source: Front Pharmacol. 2021 Jan 21;11:609756. doi: 10.3389/fphar.2020.609756 (PMC7859449; doi:10.3389/fphar.2020.609756)
Supplement: Supplementary file 1 [file datasheet1.docx]

Supplementary Material

# Supplementary Data

**Method validation for quantification of RA in rat plasma**

The method was validated for selectivity, linearity, LLOQ, accuracy, precision, recovery, matrix effect, and stability, according to the US-FDA and EMA guidance on bio-analytical method validation.

**Selectivity.** The typical chromatograms of blank plasma and blank plasma spiked with RA and IS as shown in Figure S7. The retention time of RA and IS was 1.33 and 1.45 min, respectively. No interfering peaks were found in the chromatograms of the blank plasma at retention times of RA and IS; the method showed good selectivity.

**Linearity and LLOQ.** Linear calibration curve was established with 1/x2 as the weight factor. The linear regression equation was y=0.0098x+0.00027. The method displayed good linearity in the range of 1-2000 ng/mL with the correlation coefficient (r) greater than 0.999. The LLOQ was 1 ng/mL with the signal-to-noise ratio of ˃10:1.

**Precision and accuracy.** Precision and accuracy were evaluated by the relative standard deviation (RSD) and relative error (RE) of the intraday and interday variations. The intraday variation was measured by replicate analyses (n=6) of LLOQ and QC samples on the same day, and the interday variation was determined on three consecutive days. The RSD and the RE values of the intra-/inter-day variations were all within ±15%, indicating that the precision and accuracy of the developed method were acceptable.

**Recovery and matrix effect.** The recovery was examined by comparing peak areas of QC spiked in plasma pre-extraction to that in neat solution. The recoveries ranged from 97.89% to 108.63% with an RSD from 1.40% to 6.22%, which indicates the recovery of the method was acceptable. Matrix effect was evaluated by comparing peak areas of QC spiked in plasma post-extraction to that in neat solution. As the matrix effects ranged from 90.11% to 93.90% with an RSD from 0.84%- 2.17%, it indicated that there was no significant endogenous interference. The precision, accuracy, recovery and matrix effect of RA are shown in table S1.

**Stability.** The stability was evaluated by analyzing QC samples at low and high levels (2 and 800 ng/mL) in triplicate under different conditions: 1. short-term stability: the QC samples were held 8 h at room temperature; 2. long-term stability: the QC samples were frozen 15 d at -20°C; 3.freeze and thaw stability: the QC samples were frozen 24 h at -20°C and thawed at 25°C, then refrozen at -20°C (cycle repeated three times); 4. stock solution stability: the stock solution was held 8 h at room temperature; 5. post-preparative stability: processed samples stayed 8 h at 4°C; As shown in table S2, the RSD and RE values were all within ±15%, indicating that RA maintained good stability under the storage conditions described above.

**Measurement of intracellular RA and its esters in cultured VSMC**

The samples (5 µL) were analyzed using liquid chromatography/mass spectrometry (LC-MS/MS) on an Ultimate 3000 RSLC-series system (Thermo Fisher Scientific, Sunnyvale, California, USA) coupled to a triple quadrupol mass spectrometer (AB Sciex Instruments API 4000, Concord, Ontario, Canada) equipped with an orthogonal ESI source operated in negative mode.

LC separation was performed on an Acclaim RSLC 120 C18 column (3 µm, 100 x 2.1 mm I.D., Thermo Fisher Scientific), preceded by an Acclaim 120 C18 guard cartridge (5 µm, 10 x 2 mm I.D., Thermo Fisher Scientific), at a flow rate of 0.5 mL/min and a column temperature of 25°C. The mobile phase consisted of a linear gradient mixed from 0,1% aqueous formic acid (mobile phase A) and acetonitrile (mobile phase B). The gradient ranged from 10% B at 0 min to 95% B in 30 min, purging with 95% B for 10 min, then again 10% B to equilibrate the column for 10 min before application of the next sample (total analysis time 50 min), Rosmarinic acid (RA) eluted at 8.5 min, Rosmarinic acid methyl ester (RAME) at 11.0 min, the ethyl ester (RAET) at 12.2 min, the butyl ester (RABU) at 14.8 min and the octyl ester (RAOCT) at 19.2 min.

The triple quadrupole mass spectrometer operated with the following parameters: ESI neg., IS -4500, EP -10, CUR 10, GS1 40, GS2 40, TEM 500°C, CAD 4, CEM 2500, DF 200. MRM m/z 358.9/160.8 (RA): DP -60, CE -22, CXP -7; MRM m/z 372.9/178,7 (RAME): DP -90, CE -28, CXP -27; MRM m/z 386.8/178.9 (RAET): DP -80, CE -228, CXP -33; MRM m/z 415.0/178.7 (RABU): DP -90, CE -28, CXP -31; MRM m/z 471.1/178.8 (RAOCT): DP -75, CE -32, CXP -13, dwell time for each MRM 150 ms. Selective and sensitive quantification was carried out with a linear concentration range from 0.04 ng/mL to 4000 ng/mL (correlation coefficient 0.9997).

# Supplementary Figures and Tables

## Supplementary tables

**Table S1.** Precision, accuracy, recovery, matrix effect of RA in rat plasma (n=6).

| **Concentration**  **(ng/mL)** | **Precision (RSD, %)** | | **Accuracy (RE, %)** | | **Recovery** | | **Matrix effect** | |
| --- | --- | --- | --- | --- | --- | --- | --- | --- |
|  | **Intra-day** | **Inter-day** | **Intra-day** | **Inter-day** | **Mean (%)** | **RSD (%)** | **Mean (%)** | **RSD (%)** |
| 1 | 6.24 | 7.11 | 4.84 | 0.09 |  |  |  |  |
| 2 | 2.86 | 4.42 | -1.59 | -5.69 | 113.61 | 2.48 | 90.88 | 2.17 |
| 40 | 3.23 | 4.08 | -1.81 | -4.89 | 108.89 | 1.22 | 93.90 | 1.59 |
| 800 | 2.46 | 3.78 | -0.11 | -2.01 | 106.94 | 1.23 | 90.11 | 0.84 |

**Table S2.** Stability of RA under different storage conditions (n=3).

| **condition** | **Nominal**  **Concentration**  **(ng/mL)** | **Measured**  **Concentration**  **(ng/mL)** | **RSD (%)** | **RE (%)** |
| --- | --- | --- | --- | --- |
| Short-term stability | 2 | 1.79±0.07 | 3.93 | -10.67 |
|  | 800 | 762.33±27.75 | 3.64 | -4.71 |
| Long-term stability | 2 | 2.18±0.09 | 4.12 | 8.83 |
|  | 800 | 770.67±50.01 | 6.49 | -3.67 |
| Freeze-thaw stability | 2 | 2.14±0.09 | 3.99 | 0.07 |
|  | 800 | 786.67±50.06 | 6.36 | -0.02 |
| Stock Solution Stability | 2 | 1.90±0.08 | 4.41 | -4.83 |
|  | 800 | 782.33±27.43 | 3.51 | -2.21 |
| Post-preparative stability | 2 | 1.88±0.13 | 6.85 | -5.83 |
|  | 800 | 737.00±8.00 | 1.09 | -7.88 |

## Supplementary Figures


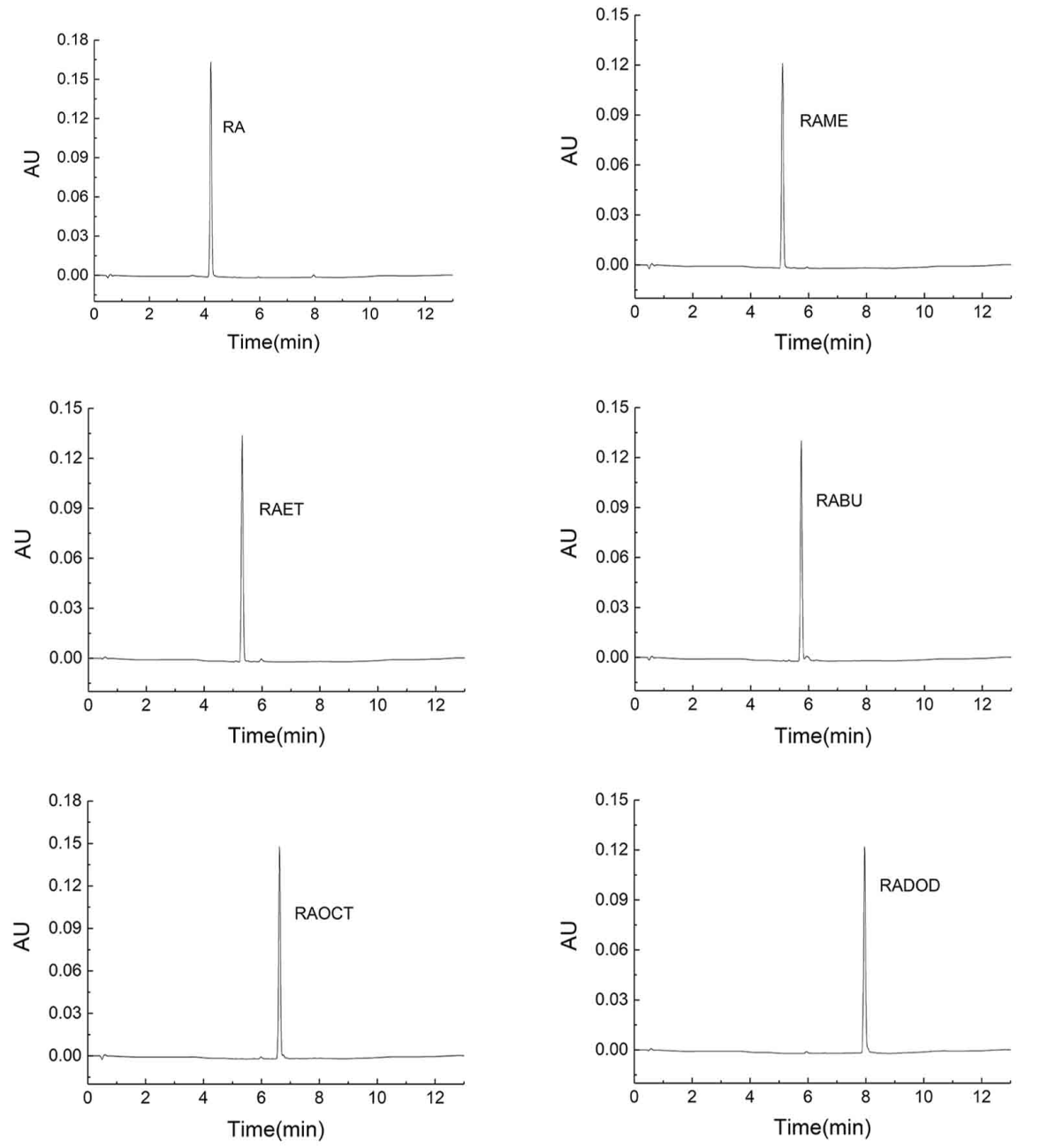


**Figure S1.** Chromatograms of RA and its alkyl esters in 50% methanol

**(A)**

**(B)**

**(C)**

**(D)**

**(E)**

**Figure S2.** Mass spectroscopic data of the synthesized RA alkyl esters. **(A)** RAME with molecular formula C19H18O8 by ESI(-)-MS at m/z 373.09299 [M–H]− (calcd. 373.09289); **(B)** RAET with molecular formula C20H20O8 by ESI(-)-MS at m/z 387.10873 [M–H]− (calcd. 387.10854); **(C)** RABU with molecular formula C22H24O8 by ESI(-)-MS at m/z 415.13998 [M–H]− (calcd. 415.13984); **(D)** RAOCT with molecular formula C26H32O8 by ESI(-)-MS at m/z 471.20276 [M–H]− (calcd. 471.20244); **(E)** RADOD with molecular formula C30H40O8 by ESI(-)-MS at m/z 527.26508 [M–H]− (calcd. 527.26504).

**Figure S3.** ^1^H NMR spectrum of RAME in DMSO-d6.

**Figure S4.** ^1^H NMR spectrum of RAET in DMSO-d6.

**Figure S5.** ^1^H NMR spectrum of RABU in DMSO-d6.

**Figure S6.** ^1^H NMR spectrum of RAOCT in DMSO-d6.

**Figure S7.** ^1^H NMR spectrum of RADOD in DMSO-d6.

**Figure S8.** Typical MRM chromatograms of RA and IS in rat plasma. **(A)** Blank plasma; **(B)** blank plasma spiked with RA and IS at LLOQ.

**RA**

**RA**

**IS**

**IS**

1.33

1.45

**(A)**

**(B)**

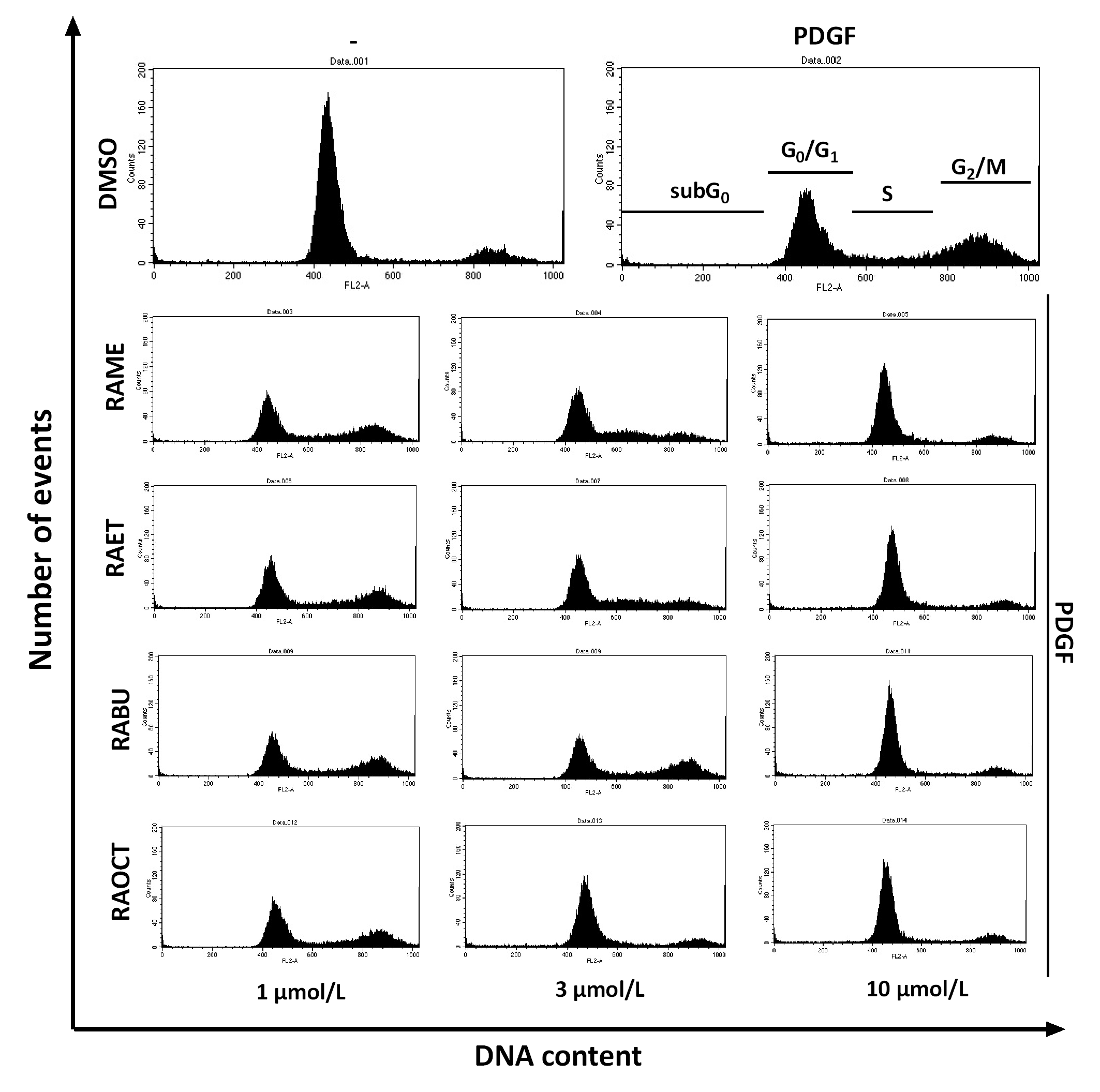


**Figure S9.** Quiescent VSMC were treated with 0.1% DMSO or increasing concentrations of RA esters as indicated for 30 min, stimulated with 20 ng/mL PDGF-BB for 16 h, and the cell cycle progression was analyzed using PI staining and flow cytometry. Histogram plots of one representative experiment are shown.
